# GABAergic cortical network physiology in frontotemporal lobar degeneration

**DOI:** 10.1093/brain/awab097

**Published:** 2021-03-12

**Authors:** Natalie E Adams, Laura E Hughes, Matthew A Rouse, Holly N Phillips, Alexander D Shaw, Alexander G Murley, Thomas E Cope, W Richard Bevan-Jones, Luca Passamonti, Duncan Street, Negin Holland, David Nesbitt, Karl Friston, James B Rowe

**Affiliations:** 1 Department of Clinical Neurosciences, Cambridge Biomedical Campus, University of Cambridge, Cambridge CB2 0QQ, UK; 2 MMRC Cognition and Brain Sciences Unit, Cambridge CB2 7EF, UK; 3 Cambridge University Hospitals, Cambridge, CB2 0QQ, UK; 4 Wellcome Centre for Human Neuroimaging, University College London, London WC1N 3AR, UK

**Keywords:** frontotemporal dementia, progressive supranuclear palsy, dynamic causal modelling, conductance-based modelling, GABA

## Abstract

The clinical syndromes caused by frontotemporal lobar degeneration are heterogeneous, including the behavioural variant frontotemporal dementia (bvFTD) and progressive supranuclear palsy. Although pathologically distinct, they share many behavioural, cognitive and physiological features, which may in part arise from common deficits of major neurotransmitters such as γ-aminobutyric acid (GABA). Here, we quantify the GABAergic impairment and its restoration with dynamic causal modelling of a double-blind placebo-controlled crossover pharmaco-magnetoencephalography study. We analysed 17 patients with bvFTD, 15 patients with progressive supranuclear palsy, and 20 healthy age- and gender-matched controls. In addition to neuropsychological assessment and structural MRI, participants undertook two magnetoencephalography sessions using a roving auditory oddball paradigm: once on placebo and once on 10 mg of the oral GABA reuptake inhibitor tiagabine. A subgroup underwent ultrahigh-field magnetic resonance spectroscopy measurement of GABA concentration, which was reduced among patients. We identified deficits in frontotemporal processing using conductance-based biophysical models of local and global neuronal networks. The clinical relevance of this physiological deficit is indicated by the correlation between top-down connectivity from frontal to temporal cortex and clinical measures of cognitive and behavioural change. A critical validation of the biophysical modelling approach was evidence from parametric empirical Bayes analysis that GABA levels in patients, measured by spectroscopy, were related to posterior estimates of patients’ GABAergic synaptic connectivity. Further evidence for the role of GABA in frontotemporal lobar degeneration came from confirmation that the effects of tiagabine on local circuits depended not only on participant group, but also on individual baseline GABA levels. Specifically, the phasic inhibition of deep cortico-cortical pyramidal neurons following tiagabine, but not placebo, was a function of GABA concentration. The study provides proof-of-concept for the potential of dynamic causal modelling to elucidate mechanisms of human neurodegenerative disease, and explains the variation in response to candidate therapies among patients. The laminar- and neurotransmitter-specific features of the modelling framework, can be used to study other treatment approaches and disorders. In the context of frontotemporal lobar degeneration, we suggest that neurophysiological restoration in selected patients, by targeting neurotransmitter deficits, could be used to bridge between clinical and preclinical models of disease, and inform the personalized selection of drugs and stratification of patients for future clinical trials.

## Teaser

Using biophysical modelling of data from controls and FTLD patients, on and off a GABAergic drug, combined with GABA spectroscopy, Adams et al. show that the potential restoration of neurotransmission in neurodegeneration depends on the degree of disruption to baseline physiology.

## Introduction

There is a pressing need for new therapeutic strategies for neurodegenerative diseases. To gain insight into the action of novel therapeutics, one requires an analytical framework that has mechanistic precision for human disease. Recent developments in the modelling of non-invasive human imaging data can facilitate such translational neuroscience. For example, dynamic causal models of neuronal network dynamics using neuroimaging data have identified the effects of genetic, auto-immune, degenerative and pharmacological perturbations of brain function.^1-4^

Here, we focus on frontotemporal lobar degeneration (FTLD), specifically patients with the syndromes of behavioural variant of frontotemporal dementia (bvFTD) and progressive supranuclear palsy (PSP). These disorders have distinctive pathology in their classical presentations: PSP presenting with dominant subcortical atrophy arising from 4-repeat neuroglial tauopathy, and bvFTD with severe frontotemporal cortical atrophy arising from 4-repeat tauopathy, 3-repeat tauopathy or TDP43 pathology. Despite these differences in pathology, they share important behavioural deficits.^5–9^ BvFTD and PSP also cause similar deficits in the neurophysiology of frontotemporal circuits, including abnormal beta-band desynchronization and connectivity, and reduced efficiency and modularity of functional circuits.^10–15^ The similarity of neurophysiological deficits contrasts with the marked difference in regional brain atrophy between bvFTD and PSP.^5,^^16-20^

We propose that similar functional deficits in the face of structural differences can be the result of impaired neurotransmission. BvFTD and PSP are associated with specific neurotransmitter deficits.^21^ In particular, FTLD pathologies cause GABAergic cell loss, synaptic loss and reductions in endogenous GABA.^22–25^ The accumulation of abnormal tau protein in FTLD has also been linked to GABAergic cell loss.^26^

In this study, we test the GABAergic hypothesis of PSP and bvFTD impairment in three complementary ways. First, we use dynamic causal modelling of magnetoencephalography (MEG) to identify local network dynamics in PSP and bvFTD, during a roving auditory oddball paradigm.^1^^,^^3^ We use this paradigm because it reveals impairments in bvFTD and PSP at the physiological level in frontal and temporal connections. We then test whether frontotemporal connectivity is proportionate to clinical severity. Second, we optimize a (conductance-based) dynamic causal model of a placebo-controlled double-blind randomized cross-over study of the GABA-reuptake inhibitor tiagabine. In healthy controls, this approach previously confirmed the predicted increase in tonic inhibition after tiagabine.^1^ Specifically, we test whether tiagabine restores the frontotemporal cortical mechanisms underlying evoked responses to unexpected sensory perturbations in PSP and bvFTD. Third, we test whether the pharmacological effect of tiagabine on the neural dynamics of frontal cortex is conditional on the degree of patients’ individual GABAergic deficit, as estimated from magnetic resonance spectroscopy (MRS) acquired at ultrahigh field (7 T).

## Materials and methods

### Experimental design

We undertook a randomized placebo-controlled double-blind crossover study to investigate the effects of tiagabine, in 32 patients (17 bvFTD, 15 PSP) and 20 age- and gender-matched healthy adults (Table 1). In keeping with the Declaration of Helsinki, written informed consent was acquired from all participants. The Cambridge Research Ethics Committee approved the study, which was exempted from Clinical Trials status by the Medicines and Healthcare products Regulatory Agency (UK). The International Standard Randomised Controlled Trial Number is 10616794 (https://www.isrctn.com/ISRCTN10616794). Participants attended two MEG sessions, 2 weeks apart. They received either 10 mg oral tiagabine or a placebo. Blood was drawn 105 min later to coincide with peak plasma levels and CNS penetration^27^ immediately prior to MEG data acquisition. A comparison across controls and patients showed evidence of equivalence for the level of tiagabine in participant serum (Bayesian independent samples *t*-test, BF_10_ = 0.301).

**Table 1:** **Group** **demographics**

	**Controls**	**PSP**	**PSP versus controls** **(BF)**	**BvFTD**	**BvFTD versus controls** **(BF)**	**PSP versus bvFTD** **(BF)**
Demographics						
Group size	20	15	–	17	–	–
Gender	M10: F10	M9: F6	n.s.	M11: F6	n.s.	n.s.
Age	67.3 (4.3)	68.8 (7.8)	1.27	61.5 (10.4)	0.239	1.20
Cognition						
MMSE	28.6 (1.4)	26.5 (3.6)	29.6	19.8 (10.3)	1.77	1.59
ACE-R						
Total (100)	95.1 (4.4)	78.0 (9.2)	1.86 × 10^3^	61.4 (27.3)	2.30 × 10^5^	1.23
Attention (18)	17.5 (0.6)	16.7 (2.3)	7.29	12.9 (6.3)	0.431	1.09
Memory (26)	24.0 (3.2)	21.9 (3.9)	1.25 × 10^3^	13.1 (8.5)	0.656	16.9
Verbal Fluency (14)	13.0 (1.0)	4.7 (2.8)	3.93 × 10^10^	4.1 (3.0)	2.68 × 10^10^	0.221
Language (26)	25.4 (1.1)	23.9 (1.8)	14.5	19.2 (7.8)	4.35	1.29
Visual spatial (16)	15.3 (1.1)	10.9 (3.7)	10.3	12.1 (4.2)	658	0.256
INECO						
Total (30)	25.4 (2.8)	17.3 (4.9)	9.90 × 10^7^	9.2 (7.0)	1.17 × 10^4^	22.8
WM index (10)	7.2 (1.3)	4.4 (2.0)	9.91× 10^4^	3.0 (2.3)	444	0.691
FAB Total (18)	17.3 (1.1)	12.1 (3.3)	2.21× 10^4^	9.2 (5.6)	3.73 × 10^4^	0.591
Hayling						
Scaled score	18.5 (1.0)	3.4 (1.9)	1.19× 10^25^	1.75 (1.1)	9.43 × 10^20^	2.40
Overall score	6.1 (0.3)	11 (6.0)	12.7	8.3 (2.9)	19.56	0.434
A + B Converted error score	2.6 (2.9)	18.7 (19.5)	5.33× 10^5^	39.7 (20.9)	199.47	2.31
Graded naming total (30)	23.8 (3.7)	20.2 (4.1)	1.21× 10^3^	12.5 (8.7)	2.32	4.18
CBI-R						
Total (170)	–	49.7 (31.0)	–	89.9 (26.0)	–	66.7
Memory and orientation (32)	–	7.3 (5.6)	–	16.9 (6.3)	–	233
Everyday skills (20)	–	11.0 (7.8)	–	11.6 (5.9)	–	0.344
Selfcare (16)	–	6.5 (5.9)	–	5.8 (5.4)	–	0.350
Abnormal behaviour (14)	–	3.3 (2.7)	–	12.3 (5.9)	–	2.34 × 10^3^
Mood (16)	–	2.2 (2.2)	–	5.2 (3.7)	–	4.69
Beliefs (12)	–	0.4 (0.8)	–	1.2 (2.1)	–	0.735
Eating habits (16)	–	3.7 (4.66)	–	9.7 (5.1)	–	19.1
Sleep (8)	–	3.1 (2.3)	–	3.0 (2.1)	–	0.340
Stereotypic and motor behaviours (16)	–	3.1 (4.4)	–	10.1 (5.3)	–	72.5
Motivation (20)	–	9.1 (6.3)	–	14.1 (4.7)	–	3.90

Cohort demographics and cognition. Gender difference was assessed using the χ^2^ test. Otherwise, Bayesian ANOVAs were used, corrected across groups for multiple comparisons. Bayes Factors (BF) are therefore presented as corrected posterior odds. Conventional thresholds for Bayes Factors represent substantial (>3), strong (>10) and very strong (>30) evidence in favour of hypotheses. CBI-R = Revised Cambridge Behavioural Inventory; F = female; M = male; WM = working memory.

Patients were recruited from tertiary referral centres within the East of England National Health Service with probable bvFTD, with or without parkinsonism^8^ or probable PSP-Richardson’s syndrome (PSP-RS^6^) including those presenting with PSP-F phenotype and subsumed under PSP-RS according the MAX-rules criteria for PSP.^28^ Healthy adults were recruited from the MRC Cognition and Brain Sciences Unit and NIHR Join Dementia Research volunteer panels, with no neurological or psychiatric illness.

In addition, participants completed a neuropsychological battery of tests commonly employed in quantifying cognitive and behavioural impairment in FTLD pathologies. These included the Revised Addenbrookes Cognitive Examination (ACE-R),^29^ the Mini-Mental State Examination (MMSE), Hayling test, INECO Frontal Screening test,^30^ Frontal Assessment Battery (FAB)^31^ and the Revised Cambridge Behavioural Inventory (CBI-R).^32^ Patients with a PSP diagnosis also had a PSP rating scale (PSPRS) examination.^6^ The group results of these tests are collated in Table 1. Note the use of Bayes factors to identify evidence in favour of the null (no group differences) as well as testing the alternate hypotheses (that the groups differ).

Neurophysiological responses were measured using MEG in a roving auditory oddball paradigm.^33^ Earpieces were used to present 75 ms binaural sinusoidal tones, with a 7.5 ms ramp up and down at the start and end of the tone, at 500 ms intervals. The tone frequency increased or decreased by 50 Hz (range 400–800 Hz) after 3 to 10 repetitions. Auditory thresholds were assessed in quiet at 500, 1000, and 1500 Hz and additionally checked in the MEG. During MEG, tones were presented at 60 dB above the population-average threshold. Participants were under continuous video monitoring: none fell asleep. The task was performed eyes-open in blocks of 5 min.

### Data acquisition and preprocessing

A 306-channel Vectorview acquisition MEG system (Elekta Neuromag) was used in an Elekta Neuromag magnetically-shielded room. This uses a sensor triplet at 102 locations (a pair of gradiometers and a magnetometer) sampled at 1000 Hz. Electroocculograms tracked eye movements vertically and horizontally and five head-position indicator coils tracked head position. A 70 channel, MEG-compatible, EEG cap (Easycap GmbH) using Ag/AgCl electrodes positioned according to the 10–20 system was used concurrently. Scalp shape was recorded with a 3 D digitizer (Fastrak Polhemus Inc) using >100 scalp points, as well as nasion and bilateral pre-auricular fiducial points.

Participants also underwent T_1_-weighted structural MRI at 3 T by Siemens PRIMSA scanner (MPRAGE sequence, echo time = 2.9 ms, repetition time = 2000 ms, 1.1 mm isotropic voxels) or at 7 T by Siemens TERRA scanner (MP2RAGE sequence 0.75 mm isotropic voxels, echo time = 1.99 ms, repetition time = 4300 ms, inversion time 1 = 840 ms, inversion time 2 = 2370 ms) at the Wolfson Brain Imaging Centre, University of Cambridge.

MEG data were first preprocessed by head position alignment and movement compensation with five head coils, using the temporal extension of Signal Space Separation with MaxFilter v2.2 (Elekta Neuromag). Bad channels were identified both manually and automatically. The Statistical Parametric Mapping toolbox (SPM12, Wellcome Trust Centre for Neuroimaging, UCL, UK) was used for subsequent preprocessing and analysis, along with custom MATLAB scripts (MATLAB 2017a, Mathworks, Natick, MA). Data were Butterworth filtered between 1 Hz and 180 Hz, epoched from −100 ms to 400 ms relative to auditory stimulus presentation. Artefact rejection was performed using electrooculogram, EEG and MEG channel thresholding, with the same thresholds applied across all groups. The deviant and standard trials were taken as the first and sixth trials of each stimulus train, respectively, averaged over frequencies. Although the task presented the same number of trials to all participants, the trial numbers used in the analysis differ between groups due to intolerance of a third block in a minority of patients, and a higher rate of artefacts in patient groups (e.g. eye blinks, occasional movements, or swallowing).

### Dynamic causal modelling: an extended canonical microcircuit model

**Figure 1:**
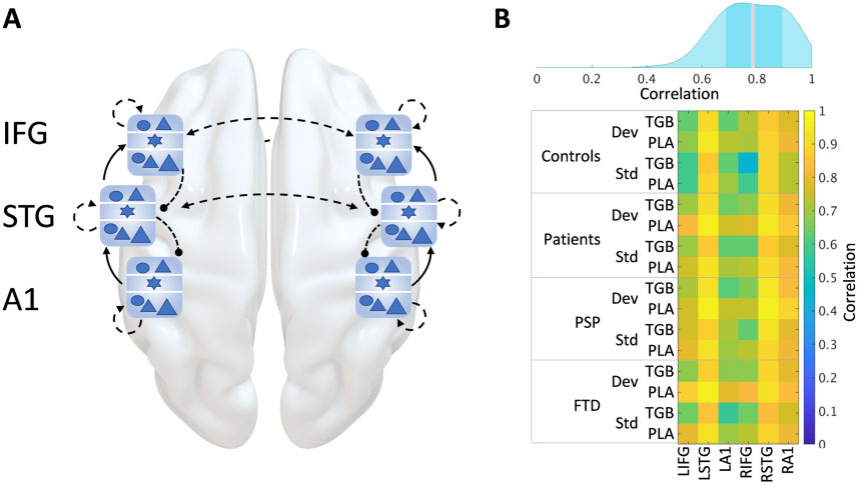
**Model schematic and accuracy.** (**A**) A schematic of the network used to model the roving auditory oddball paradigm. The six sources [bilateral primary auditory (A1), superior temporal gyrus (STG) and inferior frontal gyrus (IFG)] are each represented by a local network node of six cell populations shown in blue. These nodes are extrinsically connected with forward, backward and lateral connections (shown as solid and dashed black arrows). (**B**) Kernel density distribution (*top*) of the level of correlation between observed and modelled event related fields for all groups and conditions, with the median in red and the interquartile range shown as a darker band around the median. The correlations making up this density distribution are shown in the correlation matrix (*bottom*).

We used the extended conductance-based canonical mean field model (CMM) for evoked responses^34^ based on SPM12 (DCM10), as previously described in detail by Adams *et al*.^1^ A schematic of the model is shown in Fig. 1A. The network comprises three bilateral sources: primary auditory (A1), superior temporal gyrus (STG) and inferior frontal gyrus (IFG). The gross-anatomical model has been widely used to study the mismatch negativity response and confirmed by intracranial corticographic recordings.^35^ High density ‘whole brain’ dynamic causal models for functional MRI data are possible, but not for CMM models inverted to MEG.^36^ Although other regions of the frontal lobe are affected by bvFTD and PSP, we focus on the IFG because of the prior evidence of its role in generating the response to deviant sensory events^3^^,^^33^^,^^37^^,^^38^; its abnormal cognitive physiology in bvFTD and PSP^11^^,^^15^; and the good sensitivity of MEG to this region, in contrast to orbital and medial prefrontal cortex. The intrinsic connectivity among the neuronal populations within each source is described in detail in Adams *et al*.^1^ These sources constitute key nodes of a network generating the responses to predicted (standard) and unexpected (deviant) events.

The extended CMM model provides a more physiologically plausible parameterization of synaptic parameters, while being compatible with previous studies of this paradigm.^39–42^ Briefly, this model incorporates layer 4 stellate cells, superficial pyramidal cells, deep cortico-cortical projection neurons, deep thalamic projection neurons and separate supra- and infra-granular inhibitory interneuron populations that allow for laminar specific dynamics mediated by GABAergic neurotransmission.^42–44^ Connections between source regions were based on the fully connected models from Phillips *et al*.^37^ and Shaw *et al*.,^3^^,^^38^ originally derived from Garrido *et al*.^33^ This network formed the basis of an iterative process to find the most likely reduced model (described below). Auditory inputs to the network were parameterized using a Gaussian bump function (peak 60 ms, half-width 8 ms) to layer 4 stellates in bilateral auditory and inferior frontal cortex. The frontal cortical ‘auditory’ inputs represent the expectation of an event in the tone sequence, but not which event type. This ‘expectancy signal’ might arise from prefrontal or striatal/thalamic sources, but the source is not modelled: the inclusion of such expectancy inputs to the prefrontal cortex increases model evidence in similar auditory oddball tasks.^37^^,^^45^

The dynamic causal model (DCM) focuses on the electrophysiological response to deviant events. We have previously shown the model’s ability to recapitulate the standard, deviant and mismatched responses in healthy adults.^1^ However, the response to deviant stimuli is of particular interest, and was selected to interrogate the effects of disease and drug.

### Magnetic resonance spectroscopy

We exploited the increased signal-to-noise and spectral resolution of ultrahigh field 7 T MRS, relative to 3 T or 1.5 T MRS, using a 7 T MAGNETOM Terra scanner (Siemens Healthineers) with a 32-channel receive, single channel transmit head coil (Nova Medical). Nineteen patients (11 PSP and eight bvFTD) completed MRS, as part of a larger study.^7^ Control MRS data are from the controls described in.^7^ MR spectra were acquired serially from a right inferior frontal gyrus voxel (2 × 2 × 2 cm^3^), placed manually by the same operator using anatomical landmarks for a short-echo semi-LASER sequence^46^^,^^47^ (repetition/echo time 5000/26 ms, 64 repetitions). We used the recommended pre-scan protocol of FASTESTMAP shimming^48^ and semi-LASER water-peak flip angle and VAPOR water suppression calibration.^49^ This sequence gives reliable GABA measurements in humans *in vivo.*^50–55^ Each of the 64 individual spectral transients from each participant were saved separately. These were then corrected for effects of eddy currents, and for frequency and phase shifts using MRspa (Dinesh Deelchand, University of Minnesota, www.cmrr.umn.edu/downloads/mrspa). One patient participant was excluded for incomplete scans and movement artefacts.

A single prefrontal voxel was studied, placed over the region of prefrontal cortex in the dynamic causal model of cortical physiology. A control region of occipital lobe was also studied (reported by Murley *et al*.^7^). Additional prefrontal cortical regions were not included because of patient tolerance given the duration of the spectroscopy session.

Neurochemicals between 0.5 and 4.2 ppm, including glutamate and GABA, were quantified using LCModel (Version 6.2-3)^56^ with water scaling and a simulated basis set that included experimentally-acquired macromolecule spectra. See Supplementary Fig. 1 for illustration of the MRS Spectrum and LCModel fit for GABA and glutamate.

### Statistical analysis

For MEG, variational Bayesian model inversion and subsequent reduction identified the most likely explanation for subject-specific MEG data in terms of Gaussian posteriors over neuronal and biophysical parameters. Group and drug effects were tested using parametric empirical Bayes (PEB) analysis, based on these posterior estimates. For other data, Bayesian analysis used JASP software (JASP Team, version 0.12.2) with conventional thresholds for Bayes factors (BF) representing substantial (>3), strong (>10) and very strong (>30) evidence in favour of hypotheses. Correction for multiple tests was based on null control by fixing the prior odds to 0.5, and the posterior odds adjusted according to the number of groups being compared.^57^ Descriptive frequentist statistics were performed in MATLAB 2017a, with *P* < 0.05 considered significant.

The dynamic causal model (DCM) was inverted using source-reconstructed ERF data for all six sources for each subject for standard and deviant trials separately.^58^ In other words, we allowed the differences between standard and deviant trials to be modelled by differences in every intrinsic and extrinsic connection; enabling us to characterize group differences induced by either standard or deviant stimulus processing. Data were filtered between 0 Hz and 48 Hz. A Tukey window that did not attenuate signals between 50 ms and 350 ms after stimuli was applied. Redundant DCM parameters were removed using Bayesian model reduction at the between-subject (i.e. second) level using PEB (with a group mean). The reduced model was then used to test for group and drug effects on connectivity parameters using PEB and general linear models in the usual way.^59^ Second-level PEBs were run for each group (control, PSP and bvFTD) separately, with a third-level ‘PEB-of-PEBs’ to compare groups.^59^^,^^60^ Effects of interest were considered significant above a threshold posterior probability of >0.95.

### Data availability

The extended DCM is available at https://gitlab.com/tallie/edcm and works in conjunction with the modified SPM12 scripts provided therein. Source data may be available for non-commercial research purposes, on request from the senior author, subject to limitations to protect participant identity.

## Results

Healthy controls, PSP and bvFTD patients were age- and gender-matched (Table 1). Patients with bvFTD were impaired in comparison to healthy controls in all tests. Compared to controls, PSP patients were impaired in the INECO, FAB, Hayling and selected subscales of the ACE-R. Although bvFTD patients were impaired compared to controls on the Graded Naming Test, there was no evidence of a difference for PSP patients. Compared to PSP, patients with bvFTD performed worse on the INECO, but PSP and bvFTD were similar in terms of verbal fluency and did not differ in terms of MMSE, Hayling, ACE-R and FAB.

Following artefact rejection, the number of deviant trials were for controls 188 ± 53, patients 155 ± 55; and the number of standard trials were for controls 141 ± 42, patients 116 ± 42). Regarding MRS quality, the line-width did not differ between groups (bvFTD 13.6 ± 3.5, PSP 13.0 ± 1.9, controls 13.7 ± 1.5: Bayes factor = 4.1 in favour of the null model of no difference between groups), and group differences in Cramer-Rao lower bound were equivocal (bvFTD 15.1 ± 5.7, PSP 13.2 ± 5.7, controls 9.6 ± 1.2; Bayes factor in favour of null model = 0.4). However, SNR was lower and more variable in bvFTD (bvFTD 40.6 ± 10.0, PSP 48.2 ± 7.0, controls 53.8 ± 5.5; Bayes factor in favour of group difference = 50.7). Group-wise event-related fields are show in Supplementary Fig. 2 for each group, drug condition and region.

The following sections set out the results of dynamic causal modelling of cortical physiology, in relation to cognitive impairment, group, drug and GABA-levels. We focus on the response to deviant events.

### Dynamic causal modelling predictions accurately reflected empirical event-related fields

Using the DCM illustrated in Fig. 1A, the predicted event-related fields correlated well with the observed event-related fields (Pearson correlation coefficient: median = 0.79, interquartile range = 0.2). A distribution of these correlations can be seen in the kernel distribution plot in Fig. 1B. Supplementary Fig. 2 shows the event-related fields predicted by the model, adjacent to the observed event-related fields, for each group, drug condition and region.

There was no significant difference between the accuracy of patient and control groups. Accuracy did differ by region, with the signals from STG modelled most accurately, most likely because of the higher signal-to-noise ratio (Fig. 1B, bottom). Note that DCM furnishes parameter estimates that maximize the log model evidence (i.e. marginal likelihood), which quantifies the accuracy adjusted for complexity. In other words, the accurate fits in Fig. 1B do not represent overfitting but rather the expressivity of the DCM.

### Extrinsic connectivity findings corroborate published evidence and correlate with behavioural measures

**Figure 2:**
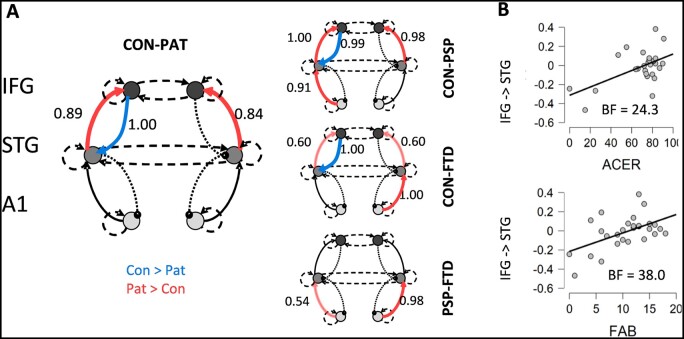
**Between-source connectivity in response to deviant stimuli: effect of group and cognitive function.** (**A**) Extrinsic connection strength difference between controls and patients in deviant trials, with blue indicating higher in controls and red meaning higher in patients (posterior probabilities are shown next to significant connections for values >0.5, and considered significant for values >0.95). Note the reduced strength of frontal lobe back projections to temporal cortex in patients. (**B**) Scatter plots show the relation between the patient scores for the ACE-R (*top*) and the FAB (*bottom*) and the strength of their fronto-temporal backward connectivity. BF = Bayes factor.

Comparing the extrinsic connections between groups, we found patient deficits in feedback frontotemporal connectivity between left IFG and left STG (Fig. 2A, posterior probability ≈1.00), consistent with previous findings.^12^^,^^61^ The loss of frontal to temporal connectivity was present in both the PSP and bvFTD groups (posterior probability = 0.99 and ≈1.00 respectively) and did not differ between PSP and FTD (Fig. 2A, bottom).

The strength of the frontal to temporal backward connections correlated with cognitive performance, measured with the ACE-R, and behavioural impairments measured with the FAB tests (Fig. 2B). These measures demonstrated strong or very strong evidence for correlations with the frontal to temporal backward connection (Bayes factors, BF_10_, ACE-R = 24.3; FAB = 38.0).

Forward connections from STG to IFG were increased, bilaterally, in patients (posterior probability = 0.89 for left and 0.84 for right), with this effect evidenced strongly in PSP (posterior probability ≈1.00 and 0.98) and weakly in bvFTD (posterior probability = 0.60 and 0.60), although the PSP versus bvFTD group difference was not significant.

### Intrinsic connectivity patterns in inferior frontal gyrus

**Figure 3:**
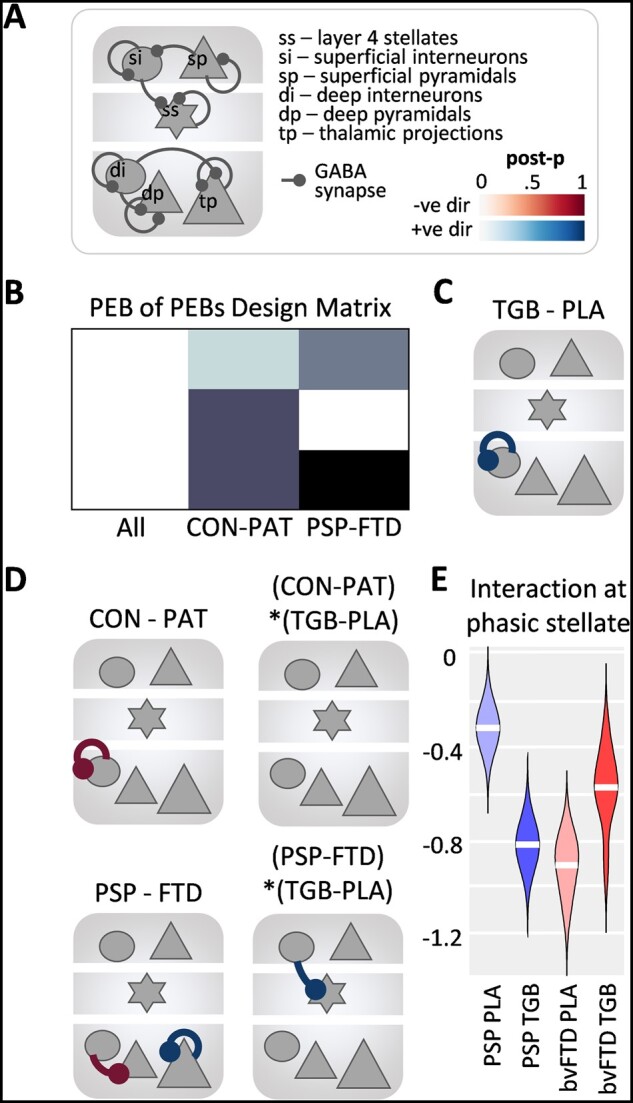
**Within-source connectivity in response to unexpected stimuli.** (**A**) Schematic showing all GABAergic synaptic connections in the model that were entered into the PEB analyses. (**B**) The PEB-of-PEBs design matrix following three second-level PEBs for the control, PSP and bvFTD groups looking at the drug condition. (**C**) The main effect of drug (tiagabine versus placebo, TGB-PLA) across all groups for deviant trials, with the connection on the deep interneurons (blue) showing high evidence for tiagabine versus placebo and no evidence for connections greater in the placebo condition (posterior probability > 0.9). (**D**) *Top row*: The main effects of controls–patients: a difference in the deep interneuron connection (red), greater for patients than controls, but no interaction with drug condition. *Bottom row*: The main effects of PSP–bvFTD: bvFTD connections from deep interneurons to deep cortico-cortical pyramidal cells are greater than PSP (red) and thalamic projection cells connection greater for PSP than bvFTD (blue). The interaction with drug condition is at the superficial interneuron to stellate cells connection (blue). (**E**) The interaction at the stellate phasic synapse for PSP–bvFTD with the drug condition showing opposing effects of tiagabine in the PSP and bvFTD groups.

Changes in extrinsic connections contextualize intrinsic or local processing within the microcircuits of regional cortical sources. Focusing on GABAergic mechanisms, we sought to explain how changes in local processing could influence the large-scale abnormalities seen in patients. With the focus on frontal cortical deficits, the following section pertains to GABAergic (intrinsic) connections in the IFG node for deviant tones. A schematic of these GABAergic connections is provided in Fig. 3A. In what follows, we characterize these differences in terms of intrinsic connectivity and their effects on the depolarization of target populations. Specifically, we can distinguish between inhibitory recurrent or self-connections and inhibitory projections from interneurons to pyramidal cells. Self-connections mediate tonic background inhibition, while intrinsic efferents to pyramidal populations can be regarded as mediating phasic inhibition.

Following a second level PEB of each group to identify drug effects, a ‘PEB-of-PEBs’ third level PEB analysis was run for all groups (Fig. 3B). Overall, tiagabine increased background inhibition in deep-layer interneurons (Fig. 3C). The joint patient group showed higher levels of tonic inhibition at these recurrent synapses when compared to the controls. But when comparing the PSP and bvFTD groups separately, there were differences at the phasic synapse onto cortico-cortical projection neurons and background (self) inhibition of cortico-thalamic projection neurons (Fig. 3D, left column).

Whereas no interactions were found between controls versus patients and the drug conditions, an interaction was found between the PSP and bvFTD groups and the drug conditions (Fig. 3D, right column). Specifically, the phasic inhibition of stellate cells showed opposite effects in the two patient groups, with the PSP cohort having high inhibition at this synaptic connection in the placebo condition, which was then decreased by tiagabine; whereas bvFTD patients had low inhibition at this synaptic connection, which increased on tiagabine (Fig. 3E).

### GABA concentration in inferior frontal gyrus explains physiological variance

**Figure 4:**
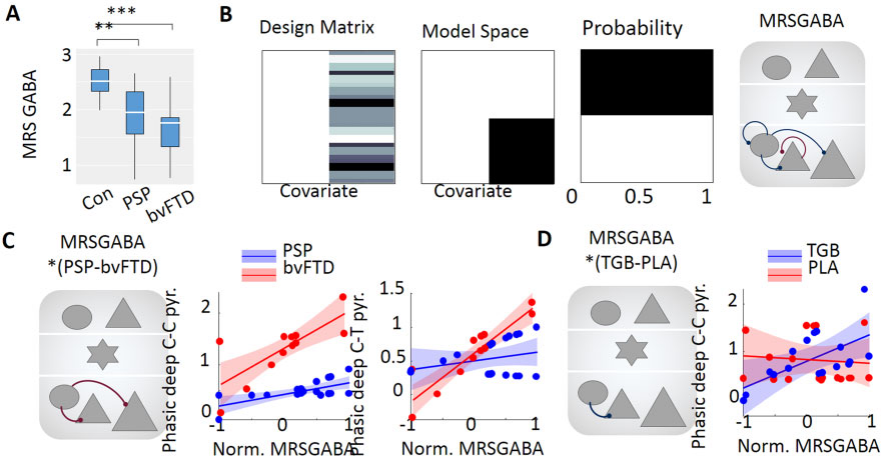
**MRS GABA levels and group interactions.** (**A**) Baseline GABA level distribution for control, PSP and bvFTD groups (Bayesian ANOVA corrected for multiple comparisons: **control versus PSP BF_10_ = 48.23; ***control versus bvFTD BF_10_ = 1862; PSP versus bvFTD BF_10_ = 0.334). (**B**) The design matrix (i.e. general linear model), model space and Bayesian model performance comparison for the model when excluding or including MRS GABA levels. *Far right*: Parameters correlating with MRS GABA levels. Evidences and colour map as in Fig. 3C. (**C**) Interaction results for MRS GABA levels and PSP–bvFTD. Each synapse shown in the node plot on the *left* is detailed in the adjacent linear regression plots. (**D**) Interaction results for MRS GABA levels and TGB–PLA. Each synapse shown in the node plot on the *left* is detailed in the adjacent linear regression plots.

The opposing responses to the drug in the two groups, and the potential dependence on initial GABA status, led us to examine the influence of baseline GABA levels. Nineteen patients (11 PSP and eight bvFTD) completed MRS. The matched control group used for spectroscopy comparison are those detailed by Murley *et al*.^7^ These 19 patients were part of a larger MRS study that confirmed reduced frontal cortical GABA concentration in PSP and bvFTD.^7^ There was very strong evidence for a difference between the control and patient groups (Bayesian ANOVA corrected for multiple comparisons^57^: PSP BF_10_ = 48.23; bvFTD BF_10_ = 1862). The contrast confirmed weak evidence of equivalence between patient groups (Fig. 4A, BF_10_ = 0.334). We therefore hypothesized that the physiological variance may be due to variations in levels and loci of GABA in the cortical microcircuit. This was explored in the context of local synaptic activity in the CMM model.

The model evidence (approximated by variational free energy) improved markedly when regional GABA was included as a between-subjects variable (Fig. 4B, posterior probability ≈1.00). For deep GABAergic synapses there was very strong evidence for a positive correlation with GABA (connections shown in Fig. 4B, far right). This dependency of GABAergic transmission (in CMM) on GABA concentration (from MRS) centred on deep-layer interneurons, affecting background inhibition of interneurons and phasic inhibition onto both cortico-cortical and cortico-thalamic projections. There was a negative effect of GABA concentration on the background inhibition of cortico-cortical projections.

The interactions between these factors were explored for the response to deviant stimuli in a separate PEB analysis (Fig. 4C–E). Interactions between GABA concentration and patient-group were identified at the deep phasic synapses onto cortico-cortical and cortico-thalamic pyramidal cell groups (Fig. 4C). This relationship is illustrated in the adjacent linear regression plots showing that the positive correlation with GABA concentration was strong in bvFTD, and weak in PSP.

An interaction between the effect of tiagabine (versus placebo) and GABA concentration was identified in the inhibitory synapses on deep cortico-cortical projection neurons (Fig. 4D), with a higher correlation evidenced between the synaptic activity and GABA concentration when patients were on tiagabine. A higher-order interaction between drug condition, GABA, and patient group was observed for the deep, tonic inhibitory synapses rather than the phasic synapses (SupplementaryFig.3).

## Discussion

The principal results of this study are that (i) biophysically informed generative models of cortical function can replicate the cortical dynamics observed in patients by MEG; (ii) the reduction in frontal to temporal backward connectivity is proportionate to cognitive performance; (iii) there is a neurochemical and functional GABAergic deficit in bvFTD and PSP. This manifests as aberrant deep inhibitory intrinsic connections, with a moderating effect of GABA concentration on the cortical physiology; and (iv) individual differences are such that the effects of tiagabine depend on GABA concentration in the frontal cortex. Taken together, these results suggest the potential for GABAergic restoration of cortical physiology in selected patients, with the ultimate goal of restoring at least in part their cognitive function.

Tiagabine was well tolerated by patients, but we stress that in this study, it was used as a pharmacological probe of cortical dynamics, not as a clinical treatment, and no clinical or behavioural outcome measures were assessed. We do not advocate its use clinically in bvFTD or PSP, but recommend that further work, including early phase clinical trials, are warranted to move from an effect on neural dynamics to potential effects on cognition and behaviour. The results we present here suggest that such early phase trials would likely benefit from participant stratification, including possibly by spectroscopic characterization.

The interest in the canonical microcircuit model used here and in Adams *et al*.^1^ goes beyond bvFTD or PSP. We used these disorders as ‘demonstrator conditions’ to test whether such models can identify clinically and pharmacologically meaningful effects at a cellular and neurochemical resolution that cannot be directly accessed *in vivo*. The critical question is not whether a disease or a drug affects neurophysiological responses, but how such an effect arises? A model can resolve mechanisms only to the level of detail specified within it: Different cellular and molecular processes may lie behind the functional deficit of a specified cell population of synapse. For this reason, the extended CMM used six cell types, separating superficial and deep cortical layers and their inhibitory populations, and thalamo-cortical connections. This was sufficient to test our principal hypothesis, but we recognize the simplification of the model with respect to heterogeneity of cell types, connectivity and neurotransmission. Finer-grained cellular, synaptic or pharmacological resolution would require more complex models (*cf*. Shaw *et al*.,^62^^,^^63^ Symmonds *et al*.^4^) demonstrated a reduction in fronto-temporal beta coherence by bvFTD, which was recapitulated in the loss of beta-band coherence and Granger causal connectivity in the non-fluent primary progressive aphasia variant of frontotemporal dementia.^61^ Moreover, both bvFTD and PSP cause a similar loss of local efficiency in the beta band, for frontal networks. We attribute these beta-frequency effects to loss of descending information to lower levels of a cortical information processing hierarchy.^61^^,^^64^ Canonical microcircuit models of phasic band-limited activity and connectivity have successfully reproduced this effect.^65^ Here, we further demonstrate that a conductance-based neuronal model can accurately generate event related field data and reveal deficits in such hierarchical extrinsic connectivity in patients. Indeed, the interregional (extrinsic) connectivity correlated with cognition and behavioural performance, linking clinical measures to a generative model level of understanding of network function.

The value of such generative models lies in their utility to predict the mechanistic nature of changes from pathology or pharmacology. There is growing evidence to support the claims of such models, drawing on the identification of the processes affected by genetic Na/Ca channelopathies, by anti-NMDA auto-immune encephalitis, and by pharmacological perturbations of brain function.^1–4^^,^^62^^,^^63^ With such diverse validation studies, the canonical microcircuit model approach promises novel insights into mechanisms of action or disease, or new candidate pharmacological targets.

The use of PEB in dynamic casual modelling—when testing for group effects—finds another application that we use for the first time in dementia research: the examination of the effects of individual differences in a neurotransmitter trait (that is to say, baseline unmedicated status) on the model optimization, and then on the individual differences in response to drug. This is conceptually related to the increase in variance explained by a covariate in a frequentist ANOVA. However, by embedding the individuals’ GABA concentration in a PEB design, we can quantify the evidence for, or against, the effect of GABA on neuronal dynamics and response to drug. Here, tiagabine’s effect on IFG in response to unexpected sensory (oddball) events was attributable to an increase to the tonic inhibition of deep inhibitory neurons, consistent with data from healthy adults.^1^ This effect on deviant trials is expected in an oddball paradigm.

Our interpretation of the changes observed in Fig. 4B is of stronger phasic inhibition deep layer neurons, whereby endogenous GABA levels could improve cortico-cortical rhythm segregation and promote coincident firing of these cells, and potentially increase bursting activity from the moderation to deep burst-firing pyramidal neurons. The latter is associated with beta-rhythm generation and the backwards propagation of information.^66^ The interaction (Fig. 4C) with patient group indicates this effect is stronger in bvFTD than PSP. This is of interest as PSP causes less frontal cortical atrophy than bvFTD^67–70^ despite a similar GABA loss and similar functional synaptic deficit.^26^^,^^71^ The effect of GABA reuptake inhibition was not confined to phasic inhibition. Indeed, the effect on tonic GABA transmission accords with the study by Dyke *et al*.^72^ study, which combined GABA spectroscopy with transcranial magnetic stimulation.

The localization of the site of drug effects, opens the way to test the physiological effects of other drugs, acting on GABA, NMDA or other principal neurotransmitters—and in other disorders. The increased tonic inhibition of these interneurons identified in our patients is considered to decrease the activity of this population, and this may be indicative of an overriding loss of deep inhibitory population activity in patients, either due to synaptic or neurotransmitter deficits.

The lack of an interaction between drug condition and the control versus patient group needs to be interpreted with caution, as there was also an interaction between the PSP versus bvFTD group and drug conditions. The drug had differential (opposing) effects between the two disorders at the phasic stellate synaptic connection, which is responsible for both activity levels of stellate cells, and the organization of information via coincident firing and rhythmic segregation.^73^^,^^74^ We speculate that this difference reflects the lesser degree of cell-loss and atrophy in PSP than bvFTD; the effects of a drug on a region where cells are present is expected to differ from the effect on a region which has sustained massive cell loss.

There are limitations to our study. First, we rely on clinical rather than pathological confirmation of the diagnosis, and therefore we cannot confirm which bvFTD cases had tau versus TDP43 pathology. Nonetheless, the accuracy of Rascovsky criteria of probable bvFTD is high,^8^ and the accuracy of PSP-RS clinical criteria is very high.^75^ Second, our study is also relatively small, with *n* ∼ 16 per group. However, note that Bayesian ‘power’ is not based on the concept of false negative rejection of the null hypothesis, but on the precision of the evidence to make an inference in favour, or against, competing hypotheses. The Bayes factors express the relative evidence between two models, and for our model selection and correlations (Figs 2–4), the evidence was very strong or decisive (BF > 10, and relevant posterior probabilities >0.95). To unpack the high order interactions into robust simple main effects may require larger group sizes. However, we note that high Bayes factors indicate not only which model is more likely, but also indicate that the precision has been sufficient to make an inference at all, rather than remain undecided (with 0.3 < Bayes factors < 3).

Third, we used DCM of the response to deviant stimuli, rather than the differential mismatch response between standard and deviant responses. We do not assume equality of network parameters between standard and deviant conditions but prioritize the parameterization of a generative model for deviant events, as an index of immediately prior GABA-dependent short-term plasticity following successive repetitions. Moreover, addition of a fourth factor of ‘trial type’ into the modelling may reduce clarity of the effects of factors of interest (disease, drug, and MRS-GABA levels), calling for the analysis of third-order interactions beyond an already complicated design.

A DCM can only resolve mechanisms to the level of detail specified in the model, in terms of its anatomical regions and the microcircuit detail within them. Models should be sufficiently complex to test the hypotheses concerned, and here we used a common gross-anatomical framework for frontotemporal interactions for change detection (auditory oddball tasks). In the frontal lobes, bvFTD also affects anterior cingulate and orbitofrontal cortex, but these regions are not well identified by MEG, because of their depth or the apposition of homologous cortex with opposite polarity on the medial surfaces. Moreover, additional regions would rapidly increase the number of parameters, risking poor convergence or local minimum solutions. Analytical solutions for Bayesian model reduction of high-density ‘whole brain’ networks are not yet possible for M/EEG, and may not be possible in view of volume conduction effects. Additional regions of MRS would also be limited by participant tolerance. To resolve the convergence of additional cellular mechanisms onto the observed physiological deficit is possible in principle, by extensions of the microcircuit model (just as our model extended from four to six cell types to separate superficial and deep cortical layers and their inhibition). However, PEB can reveal additional complexities, in terms of interactions with other measures, such as GABA. The presence of such interactions indicates that a drug treatment is likely to be selective in its benefits (or adverse effects) according to the state of an individual patient. Simple group-wise comparisons can thereby be misleading in isolation. For the GABA spectroscopy, a limitation was that only a subset of the patient group underwent MRS, due to temporal offset in the readiness of the methods. However, these analyses also draw on evidence-based Bayesian analyses, not frequentist statistics, and were sufficiently powered for the effects we observed (and the large effects sizes expected, from Murley and Rowe^21^). We also note that hidden parameters identified by DCM are interdependent, such that the examination of the influence of individual parameters can be confounded by covariance. The PEB process considers parameter covariances that would be ignored if using traditional frequentist approaches to mass-univariate inferences on parameters, and thus is preferable for hierarchical modelling.

This study did not aim to identify a cognitive benefit of group-wise treatment, nor chronic treatment effects—it is not a clinical trial. We focus instead on the identification of mechanisms of disease and drug intervention. To determine clinical efficacy requires a clinical trial, which we believe is indicated. Such trials are a pressing need for bvFTD, PSP and dementia generally.^76–78^ We propose that in PSP, FTD and other neurological and psychiatric disorders,^38^^,^^79–81^ the combination of model-based physiology and targeted psychopharmacology can provide critical evidence to reduce the risk of such trials, reducing cost, duration and failure rates of phase II-III trials.

## Supplementary Material

awab097_Supplementary_Data

## Abbreviations

ACE-R: Revised Addenbrookes Cognitive Examination
A1: primary auditory cortex
bvFTD: behavioural variant frontotemporal dementia
CMM: canonical mean field model
DCM: dynamic causal model
FAB: Frontal Assessment Battery
MEG: magnetoencephalography
MRS: magnetic resonance spectroscopy
PEB: parametric empirical Bayes
PSP: progressive supranuclear palsy
