# Supplementary material for: GABAergic cortical network physiology in frontotemporal lobar degeneration
Source: Brain. 2021 Mar 12;144(7):2135–45. doi: 10.1093/brain/awab097 (PMC8370432; doi:10.1093/brain/awab097)
Supplement: awab097_Supplementary_Data [file awab097_supplementary_data.pdf]

Supplementary Figure S1 – Magnetic resonance spectrum and LCModel.

The Figure illustrates the LCModelling of the MR spectrum, indicating the baseline correction, and two of the library spectra (for GABA and Glutamate), whose weighted contributions alongside other model-spectra in the “LCModel fit” provide a close approximation to the observed data.

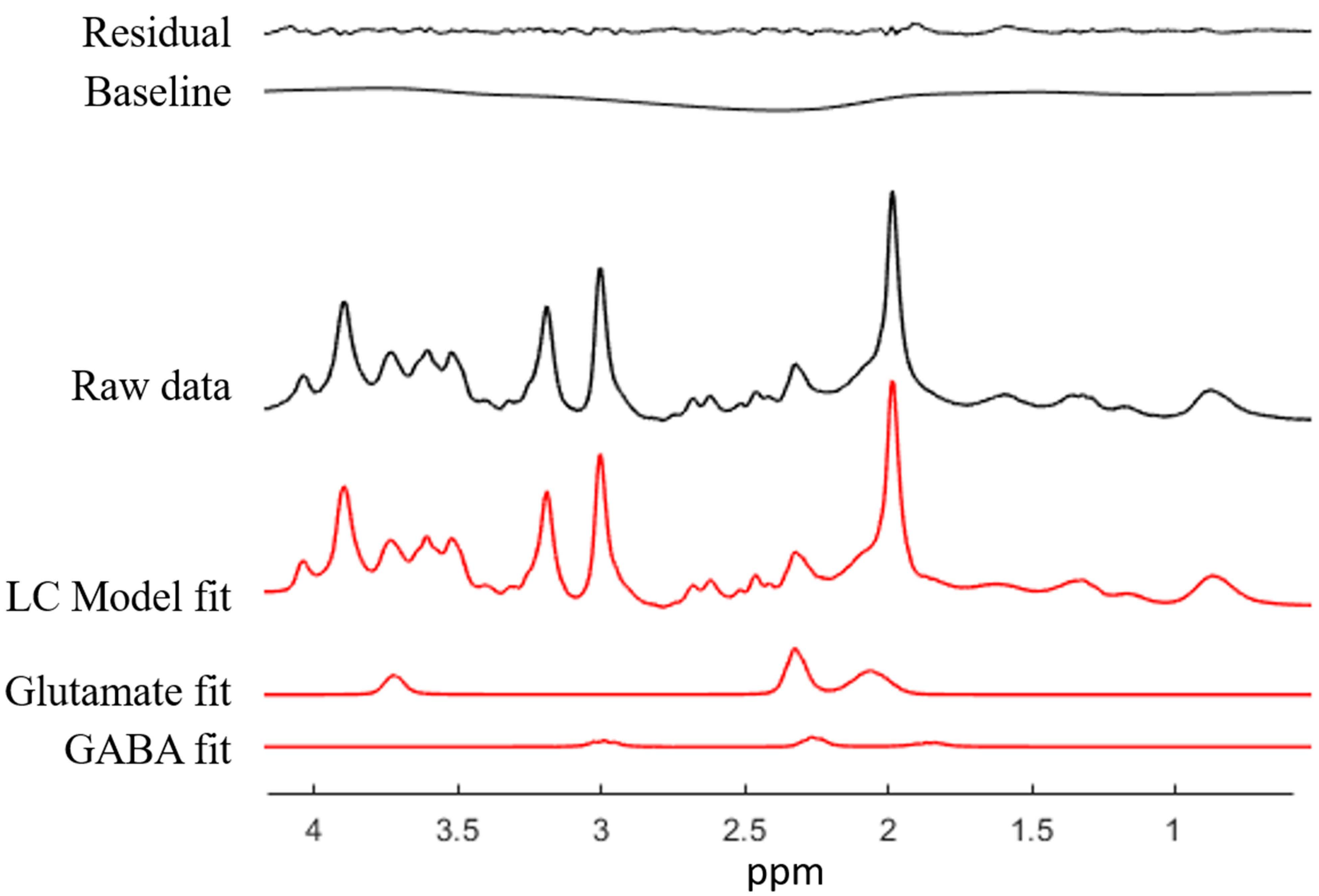
Residual
Baseline
Raw data
LC Model fit
Glutamate fit
GABA fit
4
3.5
3
2.5
2
1.5
1
ppm

Figure S1

Supplementary Figure S2 – Observed and predicted event-related fields.

Event related fields in response to deviant trials, for patients (red) and controls (blue) under placebo (upper panel) and tiagabine (lower panel) as observed (solid) and predicted (dashed) by the dynamic causal model.

Placebo

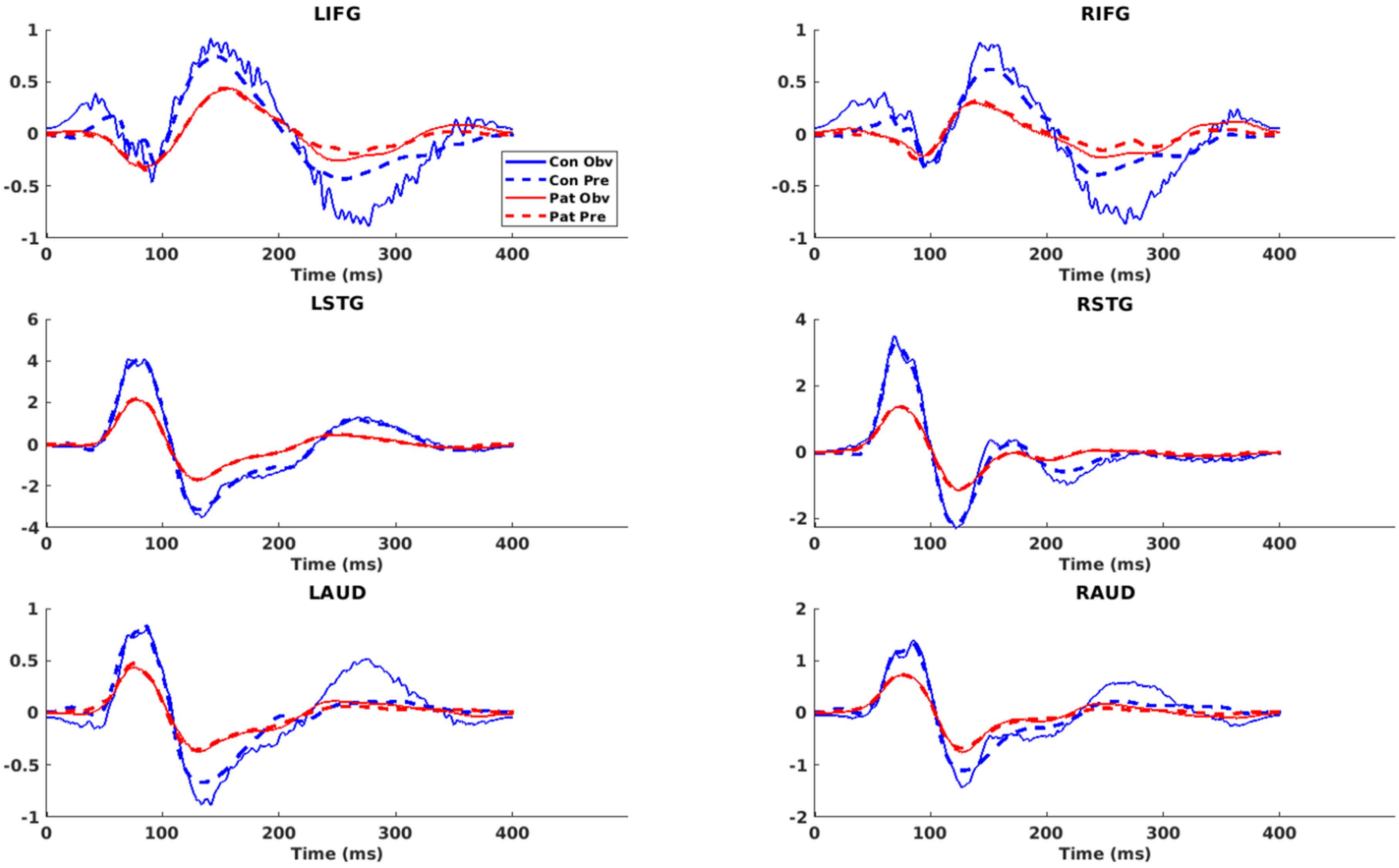

Drug

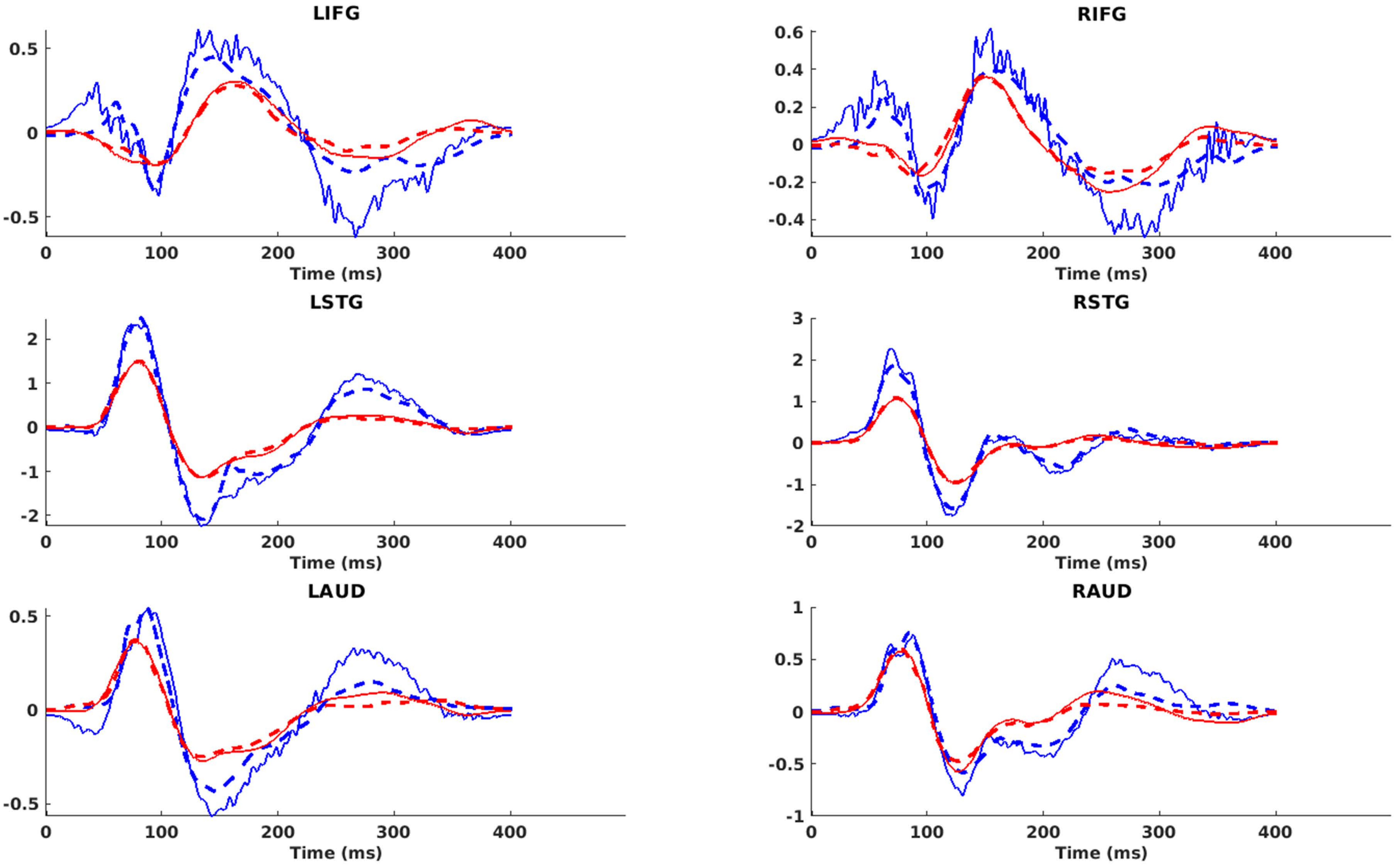

Figure S2

Supplementary Figure S3 - Interaction between GABA, group and drug.

The high-order interaction between MRS-GABA levels, PSP-bvFTD group and TGB-PLA drug effect at the deep tonic inhibitory cell connection, following PEB (left). This interaction is illustrated in the line plot (right: confidence intervals following PEB lie within the thickness of the line), which shows how the PSP and bvFTD groups have differential effects of drug on the relationship between this intrinsic connection and GABA concentration. In both patient groups, higher intrinsic GABA levels are associated with stronger inhibitory connectivity. However, the moderation of this positive relationship by tiagabine differs between PSP and bvFTD.

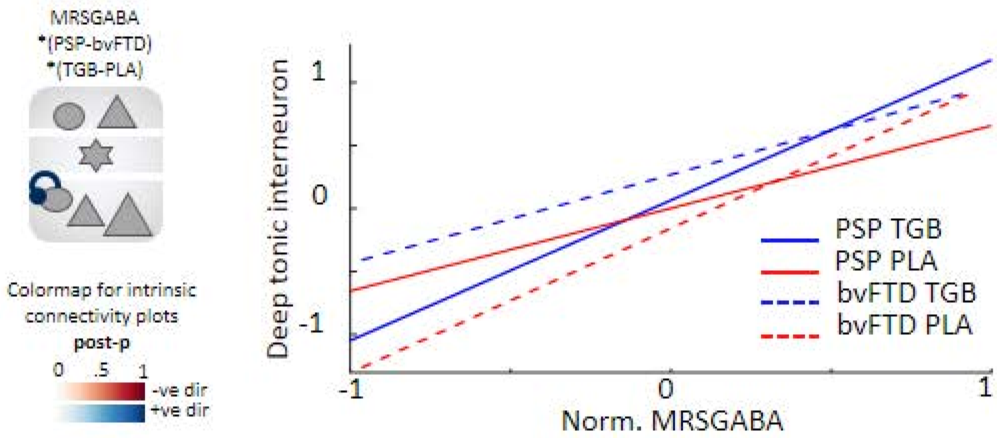
MRSGABA
*(PSP-bvFTD)
*(TGB-PLA)
Colormap for intrinsic connectivity plots
post-p
0
.5
1
-ve dir
+ve dir
Deep tonic interneuron
1
0
-1
-1
0
1
Norm. MRSGABA
PSP TGB
PSP PLA
bvFTD TGB
bvFTD PLA

Figure S3
